# Frecuencia de anticuerpos y seroconversión frente a *Rickettsia* spp. en pacientes atendidos en instituciones de salud del departamento de Caldas, Colombia, 2016-2019

**DOI:** 10.7705/biomedica.5712

**Published:** 2021-10-15

**Authors:** Jorge Enrique Pérez, Gloria Inés Estrada, Yuliana Zapata, Marylin Hidalgo, Cristian Camilo Serna, Diego Camilo Castro, Cristian González

**Affiliations:** 1 Grupo de Investigación Biosalud, Universidad de Caldas, Manizales, Colombia Universidad de Caldas Universidad de Caldas Manizales Colombia; 2 Facultad de Ciencias para la Salud, Universidad Católica de Manizales, Manizales, Colombia Universidad Católica de Manizales Universidad Católica de Manizales Manizales Colombia; 3 Grupo de Enfermedades Infecciosas, Facultad de Ciencias, Pontificia Universidad Javeriana, Bogotá, D.C., Colombia Pontificia Universidad Javeriana Pontificia Universidad Javeriana Bogotá, D.C. Colombia; 4 Grupo de Investigación Gebiome, Programa de Biología, Universidad de Caldas, Manizales, Colombia Universidad de Caldas Universidad de Caldas Manizales Colombia; 5 Departamento de Ciencias Básicas, Universidad de Caldas, Manizales, Colombia Universidad de Caldas Universidad de Caldas Manizales Colombia; 6 Sección de Admisiones, Clínica Versalles, Manizales, Colombia Clínica Versalles Manizales Colombia

**Keywords:** Rickettsia, Rickettsia rickettsii, Rickettsia typhi, Rickettsia felis, infecciones por *Rickettsia*/diagnóstico, epidemiología, factores de riesgo, Rickettsia, Rickettsia rickettsia, Rickettsia typhi, Rickettsia felis, *Rickettsia* infections/diagnosis, epidemiology, risk factors

## Abstract

**Introducción.:**

Las rickettsiosis son enfermedades zoonóticas transmitidas por artrópodos que cumplen el papel de vectores y reservorios, y cuyos síntomas son inespecíficos, por lo que su diagnóstico clínico es difícil. La inmunofluorescencia indirecta (IFI) es el método de referencia para el diagnóstico. En Colombia, ha resurgido el interés por su estudio por los casos de rickettsiosis detectados en el norte del departamento de Caldas a partir del 2001.

**Objetivo.:**

Establecer la frecuencia de anticuerpos y la seroconversión contra *Rickettsia* spp. en pacientes atendidos en instituciones de salud del departamento de Caldas, Colombia, entre 2016 y 2019.

**Materiales y métodos.:**

Se hizo un estudio de diseño cuantitativo, observacional y descriptivo, con una muestra no probabilística de 175 pacientes atendidos en diferentes municipios de Caldas, a quienes se les realizó IFI para la detección de anticuerpos en fase aguda y convaleciente contra *Rickettsia rickettsii, R. typhi y R. felis.*

**Resultados.:**

El promedio de edad de los pacientes fue de 31 años. Los municipios con mayor proporción de seropositivos fueron Belalcázar, Chinchiná, Filadelfia, La Dorada, La Merced y Manizales. El 66 % tenía mascotas y el 12 % reportó picaduras por artrópodos. Los signos y síntomas más frecuentes fueron cefalea (69,7 %), artromialgia (60 %), y fiebre (58,2 %). La seroprevalencia por IgG fue de 60 % para *R. rickettsii*, 47,9 % para *R. typhi* y 24 % para *R. felis.* Ocho pacientes presentaron seroconversión.

**Conclusión.:**

Se encontró evidencia de la circulación de rickettsias del grupo de las fiebres manchadas y del grupo del tifus asociada con casos humanos en el departamento de Caldas.

## Abstract (en)

**Introduction:**

Rickettsioses are zoonotic diseases transmitted by arthropods acting as vectors and reservoirs. Disease symptoms are nonspecific and, therefore, their clinical diagnosis is difficult. Indirect immunofluorescence (IFA) is the gold standard assay for diagnosis. The interest for conducting studies on these pathologies has resurfaced in Colombia since 2001; besides, previous studies have evidenced cases of rickettsiosis in the north of the department of Caldas.

**Objective:**

To establish the frequency of antibodies and seroconversion against *Rickettsia* spp. In patients consulting health institutions in Caldas, Colombia, from 2016 to 2019.

**Materials and methods:**

We conducted a quantitative, observational, and descriptive study on a non-probabilistic sample of 175 patients with symptoms compatible with rickettsiosis who consulted in different municipalities of Caldas, Colombia; IFA was performed to detect antibodies in the acute and convalescent phases against *Rickettsia rickettsii, Rickettsia typhi,* and *Rickettsia felis.*

**Results:**

The average age of the patients was 31 years. The municipalities with the highest proportion of seropositive cases were Belalcázar, Chinchiná, Filadelfia, La Dorada, La Merced, and Manizales; 66% of patients owned pets and 12% reported arthropod bites. The most frequent signs and symptoms were headache (69.7%), arthromyalgia (60%), and fever (58.2%). IgG seroprevalence was 60% for *R. rickettsii,* 47.9% for *R. typhi*, and, and 24% for *R. felis*. Eight patients presented seroconversion.

**Conclusion:**

We found evidence of the circulation of *Rickettsia* species from the spotted fever group and the typhus group associated with human cases in Caldas.

Colombia se ubica en el trópico, una zona endémica para distintas enfermedades infectocontagiosas, entre ellas, las rickettsiosis ^1^. La Organización Mundial de la Salud (OMS) modificó en 1998 el sistema de vigilancia para enfermedades de reporte obligatorio bajo el concepto de vigilancia sindrómica, grupo en el que se encuentran las rickettsiosis ^2^. En la semana epidemiológica 53 de 2020, el Instituto Nacional de Salud reportó 78,979 casos de dengue, 160 casos de chikungunya y 165 casos de Zika; sin embargo, no se reportaron casos de rickettsiosis ^3^, lo que se explicaría por la baja sospecha de casos y la no obligatoriedad de la notificación.

Las rickettsias son bacilos Gram negativos de crecimiento intracelular obligado y multiplicación en el citoplasma de las células endoteliales ^4^^,^^5^. Se clasifican en cuatro grupos: el grupo de las fiebres manchadas (*R. rickettsii, R. parkeri, R. ambliommatys, R. africae y R. conorii,* entre otras), el grupo del tifus (*Rickettsia prowazekii y R. typhi*), el grupo ancestral (*R. bellii y R. canadensis*) y el grupo transicional (*R. felis, R. akari y R. australis*) ^5^. Se transmiten por vectores como ácaros, garrapatas, piojos o pulgas. El ser humano se infecta cuando es picado por un vector o por contacto de la piel o mucosas lesionadas con secreciones infectadas ^6^.

Aunque las rickettsias ocurren en todo el mundo, se diagnostican poco debido a la inespecificidad de sus síntomas y a que no se distinguen bien de otras enfermedades infecciosas y no infecciosas ^7^. Los síntomas más frecuentes son fiebre, cefalea y artromialgias ^4^ y, en menor medida, náuseas, emesis y dolor abdominal, así como síntomas neurológicos con una frecuencia menor al 50 % ^6^^,^^7^. La tríada de fiebre, cefalea y exantema, descrita como clave en el diagnóstico, es irregular y, especialmente, el exantema se presenta con menor frecuencia ^4^^,^^8^. Ante el subdiagnóstico, solo una aguda percepción de síntomas «similares a la influenza» puede aumentar el grado de sospecha diagnóstica, independientemente de la exposición a los vectores, ya que la infestación con garrapatas o pulgas puede ser breve e, incluso, pasar inadvertida. Sin embargo, este factor de riesgo debe tenerse en cuenta al entrevistar al paciente ^9^.

La inmunofluorescencia indirecta (IFI) se considera la prueba de referencia para el diagnóstico de las rickettsiosis, con una sensibilidad de 83 a 100 % y una especificidad de 99 a 100 %, aunque su utilidad se ve limitada por la amplia reacción cruzada dentro de un mismo grupo de rickettsias; además, el rendimiento diagnóstico depende del momento de la toma de las muestras ^10^. La IFI *detecta anticuerpos contra rickettsias que* pueden persistir durante años después de la infección, lo que implica que un solo título de anticuerpos no es confiable para diagnosticar la enfermedad incidente. Durante la fase aguda, los resultados pueden ser negativos, pues no se han producido los anticuerpos, y por eso se requiere una segunda muestra que debe tomarse 15 a 60 días después de la primera ^11^. El aumento en cuatro veces o más de los títulos de IgG en la fase de convalecencia, se considera como un dato positivo (seroconversión) ^10^^,^^12^.

El uso de métodos moleculares para la detección de rickettsias tiene sus limitaciones. A menos que haya un proceso clínico avanzado, la detección en sangre puede ser baja, aunque el paciente esté en la fase aguda. En estos casos, se ha encontrado una mayor sensibilidad en la detección de ADN de rickettsia en tejidos ^13^^,^^14^. Los métodos diagnósticos como el cultivo no se usan con frecuencia por su grado de dificultad y la necesidad de niveles altos de bioseguridad ante el riesgo de exposición ^9^. Las rickettsias no se detectan en frotis sanguíneos ni se colorean con las tinciones convencionales y los resultados de laboratorio convencionales, como el hemograma, no sugieren la infección ^14^^,^^15^. Otros enfoques diagnósticos proponen la clasificación del paciente según el área geográfica de procedencia y la atención diferencial frente a otras enfermedades febriles ^1^.

El primer caso de la fiebre manchada de las Montañas Rocosas se registró en 1899 en Norteamérica y, en Suramérica, el primer país en reportar la enfermedad fue Brasil ^15^^,^^16^. En Colombia, esta fiebre fue inicialmente descrita por el doctor Luis Patiño Camargo en pacientes con un cuadro febril exantemático grave que se denominó “la fiebre de Tobia” por el nombre del corregimiento del municipio de Nimaima (Cundinamarca) donde se presentó el brote ^17^.

Después de un silencio epidemiológico de aproximadamente 70 años, en el 2001 en la zona rural de Ciénaga de Oro (Córdoba), se encontró una seroprevalencia del 49 % frente a rickettsias del grupo de las fiebres manchadas en los sujetos analizados y, posteriormente, en el 2005 en Villeta (Cundinamarca), se reportó una seroprevalencia del 40,3 % en personas sanas frente este mismo grupo de rickettsias ^18^^,^^19^. En las últimas dos décadas, se han informado varios brotes de *R. rickettsii* en Tobia en el 2003 y en Villeta en el 2004 ^20^; en Necoclí y Turbo (Antioquia) en el 2006 y el 2008, respectivamente ^21^^,^^22^; en Los Córdobas (Córdoba) en el 2007 ^23^ y en Uramita (Antioquia) en el 2014 ^24^. Aún persisten muchas incógnitas sobre la presencia o circulación de estos patógenos en el territorio colombiano y sobre sus respectivos vectores.

La región norte del departamento de Caldas es una zona endémica para el tifus (*R. typhi*) según los reportes del sistema de vigilancia epidemiológica del Instituto Nacional de Salud. En el 2010, este departamento reportó el mayor número de casos ^25^ y, en el 2005, un estudio con métodos serológicos demostró la presencia de tifus endémico en la región, con un 29 % en la población pediátrica y 9 % en la población adulta bajo evaluación ^26^. Entre el 2010 y el 2011, se hicieron otros dos estudios. En el primero, mediante pruebas moleculares (PCR) para la detección de rickettsias en pulgas de animales, se encontró que el 41 % de las muestras era positivo para tres genes de rickettsias *(gltA, ompB y 17kD*), identificándose en la gran mayoría de ellas la especie *R. felis*, con lo que se confirmó la endemicidad de esta *Rickettsia* en el departamento ^27^.

En el segundo estudio, se determinó la frecuencia serológica de exposición a *Rickettsia* spp. asociada con pulgas mediante IFI, y se encontró que el 17,8% de la muestra obtenida tenía IgG contra *R. felis*, el 25,2%, IgG contra *R. typhi* y el 28,7% presentaba reactividad frente a las dos rickettsias ^28^. De 26 pacientes en quienes se logró obtener la muestra de suero en fase aguda y convaleciente, dos presentaban seroconversión frente a *R. typhi*, uno frente a *R. felis*, uno tenía reactividad frente a estas dos rickettsias y cinco frente a *R. rickettsii*, con lo que se estableció que dichos pacientes presentaban una rickettsiosis activa ^28^.

En un estudio reciente en pulgas obtenidas entre el 2015 y el 2017 de perros, gatos, marsupiales y roedores en 23 municipios de Caldas, se encontró que, de 911 conjuntos de pulgas recolectadas, el 89,8% era positivo para el gen *gltA*, 46,5% para el gen *Sca5* y 96,9% para el gen *htrA* de rickettsias. Mediante análisis de polimorfismos de los fragmentos de restricción y de secuenciación, se encontraron patrones moleculares asociados con *R. felis* y *R. asembonensis*^29^.

En este contexto, el objetivo del presente estudio fue determinar la frecuencia de anticuerpos y de seroconversión frente a diferentes especies de rickettsias del grupo de las fiebres manchadas, del grupo del tifus y del grupo transicional en pacientes que ingresaron a los servicios de urgencias de diferentes instituciones prestadoras de servicios de salud del departamento de Caldas, entre enero de 2016 y diciembre de 2019.

## Materiales y métodos

Se hizo un estudio cuantitativo, observacional y descriptivo de corte longitudinal, con una muestra constituida por 175 pacientes que consultaron al servicio de urgencias en instituciones de Caldas en el periodo comprendido entre enero del 2016 y diciembre del 2019 por síntomas inespecíficos, y cuyas manifestaciones no fueron explicadas por otra causa.

Con base en los criterios de inclusión, se seleccionaron aquellos pacientes de zona urbana o rural, de cualquier edad, cuyo sitio de procedencia fuera el departamento de Caldas y que hubieran consultado por presentar uno o varios de los siguientes síntomas: fiebre, cefalea, artromialgia, dolor abdominal, náuseas, emesis, diarrea, tos o erupción cutánea. Se excluyeron aquellos que, aun presentando la sintomatología, tenían o podían tener una mayor probabilidad de otro tipo de diagnóstico. El registro de los datos estuvo a cargo del médico de la atención inicial en un cuestionario diseñado para tal fin.

Se utilizaron 40 variables divididas en siete subgrupos (cuadro 1 suplementario), con el fin de orientar los resultados hacia el cumplimiento de los objetivos. Se tuvieron en cuenta las variables sociodemográficas, las características de la casa, la presencia de mascotas (perros, gatos, otros), el contacto con animales domésticos como bovinos y equinos, la presencia de ratones y zarigüeyas, la frecuencia de picaduras por pulgas y garrapatas, los signos y síntomas, y la presencia de IgG contra *R. rickettsii*, *R. typhi* y *R. felis.*

La recolección de las muestras y de la información se hizo en los hospitales locales de 22 de los 27 municipios de Caldas. Los pacientes aceptaron participar en el estudio mediante la firma de un consentimiento informado; se les tomó una muestra de suero en la fase aguda de la enfermedad y se les citó para la toma de una segunda muestra al cabo de quince días (fase convaleciente).

### Inmunofluorescencia indirecta

Para la detección de IgG frente a rickettsias del grupo de las fiebres manchadas (antígeno: *R. rickettsii*) y de rickettsias del grupo del tifus (antígeno*: R. typhi*), se utilizaron pruebas de IFI de la marca Focus (Cypress, California; USA); para la detección de IgG frente a rickettsias del grupo transicional (antígeno, *R. felis*), se usaron pruebas de IFI de marca Fuller (Fullerton, California, USA).

En el procesamiento de todas las muestras se siguieron los protocolos de los fabricantes. Se consideró que la muestra era positiva cuando presentaba una fluorescencia igual o mayor que la del control positivo a una dilución de 1:64. Con el fin de establecer si había seroconversión, en aquellas muestras obtenidas en la fase aguda y convaleciente que habían sido positivas para IgG en el ensayo inicial, se cuantificaron las concentraciones de este anticuerpo haciendo diluciones seriadas con un factor de dilución de 2. La lectura de los ensayos IFI estuvieron a cargo de un solo observador; como controles positivo y negativo, se utilizaron los suministrados por los estuches (controles individuales positivos con IgG frente a *R. rickettsii, R. typhi y R. felis*); el conjugado provenía de las marcas de las pruebas utilizadas (anti-IgG humana de cabra marcada con fluoresceína específica para las cadenas gamma).

En todas las muestras obtenidas en las fases aguda y convaleciente, se determinó de manera simultánea la presencia de IgG frente a las tres rickettsias que hicieron parte del estudio.

### Análisis estadístico

La información se tabuló en una base de datos en Excel que luego se exportó al programa Epi-Info 7.2 empleado en el análisis de los datos mediante la aplicación de estadística descriptiva.

Se aplicaron medidas de tendencia central, de dispersión y posición, y las variables cualitativas se expresaron en distribuciones de frecuencias absolutas y relativas.

Para establecer el grado de asociación entre los factores de riesgo y las frecuencias de IgG, se empleó la prueba de ji al cuadrado con un valor de p<0,05 y se calculó la razón de prevalencias con sus respectivos intervalos de confianza del 95%.

Para el cálculo de la tasa de prevalencia de rickettsiosis, se tomó la población con resultados positivos de IgG en la primera muestra y el total de habitantes del departamento para el 2019 (923.472 habitantes) según los datos del DANE.

Para establecer la frecuencia de casos activos de rickettsiosis, se analizaron los títulos de IgG obtenidos en las fases aguda y convaleciente de todos los pacientes con fiebre, y se estableció que la enfermedad estaba presente cuando se registraba un aumento, por lo menos, de cuatro veces en el título de anticuerpos en la segunda muestra ^30^.

El control de los sesgos de selección y de información se hizo en la fase de diseño, especificando los criterios de inclusión y exclusión, y generando un formato de variables por registrar para que las unidades de análisis fueran lo más homogéneas posible. Sin embargo, se pudo presentar sesgo de información, ya que la medición de las variables se basó en los datos registrados en los formatos y, en ellos, algunas de estas carecían de información.

### Aspectos éticos

El estudio fue aprobado por el Comité de Ética Médica de la Universidad de Caldas mediante el acta N^o^ 006 del 2014. Todos los participantes firmaron el consentimiento informado. Según la Resolución 8430 del 4 de octubre de 1993 expedida por el Ministerio de Salud, este estudio se clasificó como de bajo riesgo, ya que la probabilidad de que los participantes sufrieran algún tipo de daño como consecuencia del estudio era baja. Por otra parte, el riesgo de contaminación del medio ambiente fue mínimo, pues el manejo de las muestras y los reactivos se ajustó a la normatividad vigente.

## Resultados

### Datos sociodemográficos

Se obtuvo una muestra de 175 personas pertenecientes a 22 de los 27 municipios del departamento de Caldas; no se recolectaron muestras en los municipios de Victoria, Supía, Aranzazu, Palestina y Marulanda (cuadro 1).

**Cuadro 1.** Distribución de la frecuencia de IgG contra *Rickettsia* spp. según el municipio

Municipio	N	**Frecuencia de IgG frente a *Rickettsia* spp.**	Positivos n (%)
Aguadas	5	2 (40)
Anserma	6	4 (66,7)
Belalcázar	24	22 (91,7)
Chinchiná	5	4 (80)
Filadelfia	7	5 (71,4)
La Dorada	12	9 (75)
La Merced	2	2 (100)
Manizales	10	8 (80)
Manzanares	1	0 (0)
Marmato	3	1 (33,3)
Marquetalia	20	9 (45)
Neira	20	17 (85)
Norcasia	20	10 (50)
Pácora	3	1 (33,3)
Pensilvania	4	4 (100)
Riosucio	1	1 (100)
Risaralda	5	4 (80)
Salamina	3	2 (66,7)
Samaná	6	4 (66,7)
San José	7	5 (71,4)
Villamaría	3	2 (66,7)
Viterbo	8	5 (62,5)
Total	175	121 (69,1)

La edad media de la población de estudio fue de 31 años, con una desviación estándar de 22 años. Los rangos de edad con mayor proporción de personas en orden descendente fueron los de 0 a 10 años (n=35), de 11 a 20 años (n=33), de 41 a 50 años (n=29) y de 31 a 40 años (n=23). En cuanto a la distribución por sexos, 99 (56,6%) eran hombres y 76 (43,4%), mujeres. La cantidad de personas procedentes del área urbana y de la rural fue similar (87 y 86, respectivamente) y la mayoría vivía en casas de material (n=129); 118 tenían mascota, predominantemente perros (n=39) y gatos (n=22), y 55 personas tenían ambas mascotas; 37 personas tenían contacto con equinos y 44 con bovinos (cuadro 2). No se encontraron diferencias estadísticas al relacionar la presencia de anticuerpos frente a *Rickettsia* spp. y el factor de riesgo según el valor de p y el intervalo de confianza de 95 %.

**Cuadro 2.** Distribución de la frecuencia de IgG contra *Rickettsia* spp. según la presencia de factores de riesgo

Variables		N	**lgG contra *Rickettsia*** **spp. (%)**	P	RP	lC_95%_
Edad (años)	0-10	35	19 (54,3)	0,049	0,75	0,55-1,04
	11-20	33	21 (63,6)	0,50	0,91	0,69-1,21
	21-30	22	17 (77,3)	0,36	1,14	0,89-1,48
	31-40	23	17 (73,9)	0,57	1,09	0,84-1,42
	41-50	29	22 (75,9)	0,36	1,13	0,89-1,43
	51-60	13	8 (61,5)	0,57	0,89	0,57-1,39
	>60	20	16 (80)	0,25	1,19	0,93-1,52
Sexo	M	99	69 (69,7)	0,86	1,02	0,83-1,25
	F	76	52 (68,4)			
Procedencia	Urbana	87	63 (72,4)	0,35	1,09	0,90-1,34
	Rural	86	57 (66,2)	0,42	0,92	0,76-1,13
	Sin dato	2	1 (50)	NA	NA	NA
Tipo de construcción	Material	129	90 (69,8)	0,97	1,00	0,80-1,25
	Bahareque	31	21 (66,2)	0,79	0,97	0,74-1,25
	Madera	10	6 (60)	NA	NA	NA
	Otro	5	5 (100)	NA	NA	NA
Mascotas	Sí	118	78 (66,1)	0,21	0,87	0,71-1,07
	No	57	43 (75,4)			
	Perros	39	24 (61,5)	0,47	0,82	0,49-1,38
	Gatos	22	17 (77,3)	0,23	1,74	0,69-4,38
	Perros y gatos	55	35 (63,6)	0,60	0,90	0,60-1,33
	Otras	2	2 (100)	NA	NA	NA
Contacto con bovinos	Sí	44	29 (65,9)	0,59	0,93	0,-1,19
	No	142	92 (70,2)			
Contacto con equinos	Sí	37	27 (73)	0,58	1,07	0,85-1,34
	No	138	94 (65,9)	0,58	1,07	0,85-1,34
Picadura de garrapatas	Sí	7	6 (85,7)	NA	NA	NA
	No	167	115 (68,9)			
	No sabe	1	0 (0)			
Picadura de pulgas	Sí	23	16 (69,6)	0,98	1,00	0,75-1,35
	No	152	105 (69,1)			
Presencia de zarigüeyas	Sí	50	34 (68,0)	0,83	0,97	0,78-1,22
	No	125	87 (69,6)			
Presencia de ratas y ratones	Sí	86	58 (67,4)			
	No	89	63 (70,8)	0,63	0,95	0,78-1,16

NA: sin datos, ya que el número de muestras fue menor o igual a cinco; RP: razón de prevalencias; IC: intervalos de confianza

### Frecuencia de inmunoglobulina G para rickettsias en sueros obtenidos en la fase aguda (primera muestra)

Se procesaron 175 muestras de suero obtenidas en la fase aguda para la detección de IgG contra rickettsias del grupo de las fiebres manchadas (antígeno: R. *rickettsii*) y del grupo del tifus (antígeno: R. *typhi*). Se utilizaron 171 muestras para la detección de IgG frente a rickettsias del grupo transicional (antígeno: *R. felis*). El 69,14 % (121 pacientes) resultó positivo para alguna de las tres especies, 105 (60,0 %) fueron positivos para rickettsias del grupo de las fiebres manchadas; 83 (47,3 %) para el grupo del tifus, y 42 (24 %) para el grupo transicional; 18 muestras (10,28 %) fueron positivas exclusivamente para el grupo de las fiebres manchadas, tres (1,7 %) para el del tifus, y 12 (6,97 %) para el transicional (cuadro 3).

**Cuadro 3.** Frecuencia de inmunoglobulina G contra *Rickettsia* spp. (grupo de las fiebres manchadas, grupo de tifus y grupo transicional) en muestras obtenidas en la primera visita de pacientes (fase aguda) atendidos en diferentes instituciones de salud en el departamento de Caldas

Variable	Categoría	Frecuencia absoluta	Frecuencia relativa (%)
IgG contra cualquier *Rickettsia* spp.	Positivo	121	69,1
	Negativo	54	30,9
	Total	175	100,00
IgG contra rickettsias del grupo de las fiebres manchadas (antígeno: *R. rickettsii*)	Positivo	105	60
	Negativo	70	40
	Total	175	100,00
Número de muestras positivas solo contra rickettsias del grupo de las fiebres manchadas		18	10,3
IgG contra rickettsias del grupo tifus (antígeno: *R. typhi*)	Positivo	83	47,4
	Negativo	92	52,6
	Total	175	100,00
Número de muestras positivas solo contra rickettsias del grupo de tifus		3	1,7
IgG contra rickettsias del grupo transicional (antígeno: *R. felis*)	Positivo	42	24,4
	Negativo	129	75,6
	Total	171	100,00
Número de muestras positivas solo contra rickettsias del grupo transicional		12	6,98

### Frecuencia de IgG en las muestras obtenidas en la fase aguda según los datos sociodemográficos

La frecuencia de muestras positivas para IgG contra *Rickettsia* spp. en los diferentes grupos de edad fue del 54 al 80 %, con un mayor porcentaje en las personas mayores de 60 años, seguidas de aquellas entre los 21 y los 30 años, los 41 y los 50, y los 31 y los 40 años (cuadro 2). La seropositividad por rickettsias del grupo de las fiebres manchadas estuvo entre el 42,9 y el 73,9 %, siendo mayor en el grupo de 31 a 40 años. Para el grupo del tifus, la seropositividad varió entre el 34,3 y el 60 %, con mayor frecuencia en los mayores de 60 años. Para las rickettsias del grupo transicional, la frecuencia de anticuerpos osciló entre el 13 y el 38,5 %, con mayor frecuencia en el rango de 51 a 60 años; en los tres análisis se observó cómo la frecuencia de IgG contra *R. rickettsii* y *R. typhi* aumentó con la edad, en tanto que, contra *R. felis*, este comportamiento no fue igual. No se encontró significación estadística al analizar dichas variables (no se presentan los datos).

Solamente Marmato, Manzanares y Pácora tuvieron pacientes sin anticuerpos, o con baja frecuencia; los otros municipios presentaron frecuencias entre el 33,3 y el 100 % (cuadro 1). La mayor frecuencia de IgG contra *R. rickettsii* se encontró en los municipios de Belalcázar, Chinchiná, Filadelfia, La Merced, Manizales, Neira y Pensilvania, con frecuencias relativas entre el 70 y el 100 %; en los municipios de Belalcázar, Chinchiná, La Merced, Neira y Pensilvania, la frecuencia de anticuerpos contra *R. typhi* estuvo entre el 60 y el 100 %, en tanto que, contra *R. felis*, varió entre un 50 y un 67 % en los municipios de Pensilvania, Risaralda, Salamina y Viterbo (no se muestran los datos).

En cuanto al sexo, la frecuencia de anticuerpos contra *Rickettsia* spp. fue levemente mayor en los hombres (69,7 %) que en las mujeres; además, el 72,4 % de los pacientes positivos procedían de la zona urbana. Otros factores de riesgo analizados, como el tipo de construcción, la tenencia de mascotas, el contacto con bovinos o equinos, las picaduras de pulga, el contacto con garrapatas, y la presencia de zarigüeyas y roedores, se asociaron con gran frecuencia a muestras positivas (títulos mayores o iguales a 64) para IgG en la fase aguda, aunque sin significación estadística (cuadro 2).

### Signos y síntomas indicativos de rickettsiosis

Con el fin de determinar la frecuencia de personas con síntomas indicativos de rickettsiosis, se tuvo en cuenta la frecuencia de presentación de los siguientes síntomas: fiebre, cefalea, artromialgias y erupción cutánea. Del total de 175 pacientes, 86 tuvieron fiebre y cefalea; 60, fiebre, cefalea y artromialgias, y 20 presentaron los cuatro síntomas.

### Variación en el título de IgG entre los sueros de las fases aguda y convaleciente

En 47 de los 102 pacientes con fiebre asociada a otros síntomas, se obtuvo la segunda muestra de suero (fase convaleciente); en ocho, se observó un aumento en el título de anticuerpos en la fase de convalecencia superior o igual a cuatro diluciones con respecto al título inicial. En tres pacientes se detectó aumento de las IgG contra rickettsias del grupo de las fiebres manchadas (*R. rickettsii*), en uno, contra las del tifus (*R. typhi*) y, en tres, contra las del transicional (*R. felis*). Un paciente exhibió seroconversión con el antígeno de las fiebres manchadas y del grupo del tifus, pero no se pudo determinar qué antígeno generó un mayor título de anticuerpos en la seroconversión debido a la similitud de ambos títulos (256 y 128, respectivamente) (cuadro 4).

**Cuadro 4.** Pacientes con seroconversión para IgG contra antígenos del grupo de las fiebres manchadas (antígeno: *R. rickettsii*), del grupo de tifus (antígeno: *R. typhi*) y del grupo transicional (antígeno: *R. felis*)

IgG							
Número de pacientes	Procedencia	*R. rickettsii* Fase aguda	***R. rickettsii* Convalecencia**	*R. typhi* Fase aguda	***R. typhi* Convalecencia**	*R. felis* Fase aguda	***R. felis* Convalecencia**	Signos y síntomas
30	Belalcázar	1:128	1:1.024	1:128	1:128	1:128	1:128	Fiebre, cefalea, artromialgias
32	Belalcázar	1:256	1:2.048	1:512	1:512	1:512	1:512	Fiebre, cefalea, erupción cutánea, artromialgias
149	Salamina	<1:64	1:256	<1:64	<1:64	<1:64	<1:64	Fiebre, cefaleas, artromialgias, náuseas y vómito, dolor abdominal
118	Norcasia	<1:64	1:256	<1:64	1:128	<1:64	1:64	Fiebre, erupción cutánea
42	Filadelfia	1:1.024	1:1.024	1:128	1:512	<1:64	<1:64	Fiebre, cefalea, tos
14	Belalcázar	<1:64	<1:64	<1.64	<1:64	<1:64	1:256	Fiebre, cefalea, erupción cutánea, artromialgias, dolor abdominal
33	Belalcázar	1:1.024	1:1.024	1:64	1:64	<1:64	1:128	Fiebre y cefalea
160	San José	<1:64	<1:64	<1:64	<1:64	<1:64	1:128	Fiebre, cefalea, artromialgia, hepatomegalia, tos, diarrea, náuseas y vómito

La procedencia de las personas que presentaron seroconversión correspondió a los siguientes municipios: Belalcázar (4 casos), Salamina, Filadelfia, Norcasia y San José (un caso en cada municipio); seis procedían de la zona rural, siete tenían mascota (perros y gatos); cinco no tenían contacto con bovinos y seis con equinos, y cinco reportaron la presencia de zarigüeyas en los alrededores de sus casas (cuadro 4).

Los síntomas acompañantes de la fiebre en estos pacientes fueron cefalea (siete pacientes), artromialgia (cinco), erupción cutánea (tres), dolor abdominal y tos (dos pacientes, respectivamente), hepatomegalia, diarrea, náuseas y vómito (cada uno en un paciente) (cuadro 4).

## Discusión

El diagnóstico de las rickettsiosis plantea un reto para el médico, debido a la inespecificidad de los síntomas y al acceso limitado a las pruebas diagnósticas. En este estudio, la frecuencia de anticuerpos asociada con cualquiera de los tres grupos de rickettsias en Caldas fue de 69,14%, (60,0%, 47,4% y 24,6%, respectivamente).

Estos resultados indican una alta frecuencia de exposición a diferentes especies del género *Rickettsia* en todo el departamento, lo cual es un parámetro clínico importante para el diagnóstico de las enfermedades febriles de origen desconocido. Las incidencias que han reportado países como Estados Unidos varían desde cero casos por cada millón de habitantes en Connecticut, por ejemplo, a 128 casos por millón de personas por año en Arkansas ^31^; se han encontrado seroprevalencias elevadas con tasas de mortalidad bajas, de 1 a 4 %, que se explican por la inespecificidad de la IFI y porque la enfermedad es causada por rickettsias menos patógenas que *R. rickettsii*, cuyos anticuerpos reaccionan de manera cruzada con antígenos de esta especie ^13^. Se cree que hasta el 30 % de los datos reportados como seropositivos para R. rickettsii puede deberse a infecciones por *R. parkeri*, aunque también se puede presentar reacción cruzada con *R. massiliae, R. amblyommatis* y *R. akari*^13^^,^^32^. En estudios en Colombia, se han encontrado seroprevalencias para rickettsias del grupo de las fiebres manchadas que varían entre 2,7 % en Necoclí (Antioquia) y 79,2 % en las veredas La Alianza, Cabuyal y Quilcace del municipio del Tambo (Cauca) ^33^^,^^34^.

Se han hecho varios estudios en el norte de Caldas para detectar anticuerpos contra *R. typhi* y *R. felis*: en el 2005, se obtuvieron 120 muestras de suero de pacientes con manifestaciones clínicas indicativas de rickettsiosis, 38 (31,7 %) de las cuales fueron positivas para IgG ^26^. En otro estudio de seroprevalencia en la misma región entre el 2010 y el 2011, se encontró una frecuencia de anticuerpos contra *R. typhi* y *R. felis* de 71,7 % ^28^. Aun cuando en el presente estudio se detectaron además anticuerpos contra *R. rickettsii*, la seropositividad fue muy similar a la reportada en el último estudio; esta elevada frecuencia se explicaría por la reacción cruzada entre especies de rickettsias o a la presencia de algunas pertenecientes al grupo de las fiebres manchadas que no han podido ser identificadas.

Hubo seroconversión en tres pacientes para rickettsias del grupo de las fiebres manchadas, en uno para rickettsias del grupo del tifus y en tres para rickettsias del grupo transicional. El número de personas con seroconversión frente a rickettsias del grupo de las fiebres manchadas fue inferior al registrado en el norte del Caldas entre el 2010 y el 2011 (cinco personas) ^28^, en tanto que frente al grupo del tifus fue mucho menor a la observada en el 2005 y entre el 2010 y el 2011 (12 y 3 personas, respectivamente) ^26^^,^^28^. Esta disminución en la frecuencia podría indicar una baja exposición de las personas a las picaduras de pulgas del género *Xenopsilla cheopis*, artrópodo que se encontr*ó* con poca frecuencia en roedores sinantrópicos y marsupiales en un estudio reciente en el departamento de Caldas, en el cual se obtuvieron diferentes especies de pulgas de animales silvestres y domésticos para establecer la presencia de rickettsias trasmitidas por pulgas en dichos artrópodos ^29^. En cuanto a la frecuencia de anticuerpos contra *R. felis*, el estudio mencionado encontró una gran frecuencia de material genómico de esta rickettsia en las muestras de ADN obtenidas de diferentes pulgas recolectadas. Este hallazgo respalda los resultados obtenidos en la seroprevalencia (24,4%) y el porcentaje de seroconversión para esta especie (6,3%). A pesar de ello, el papel de esta especie de rickettsia en la producción de manifestaciones clínicas no se ha establecido con total certeza ^35^.

En un estudio en tres municipios del Urabá antioqueño (Turbo, Necoclí y Apartadó), se estableció una seroprevalencia contra rickettsias del grupo de las fiebres manchadas del 20,1%, con seroconversión en seis de los 220 pacientes analizados (2,7 %) ^33^. En otro estudio reciente en el Urabá antioqueño, se demostró una seropositividad frente a *R. rickettsii* del 40,44%, con seroconversión en seis pacientes (6,7%) (36). Los resultados obtenidos en los sueros pareados en ambos estudios son similares a los observados en este, pero no así la seroprevalencia, que fue mayor y similar a la obtenida en el municipio de Villeta (Cundinamarca) entre el 2011 y el 2013 ^37^ y menor a la observada en el municipio del Tambo (Cauca) ^34^. Estos hallazgos son un indicio de la circulación en el territorio colombiano de diferentes especies de rickettsias asociadas con el grupo de las fiebres manchadas. La frecuencia de la picadura por garrapatas en el presente estudio fue muy baja (4%) en contraste con la reportado en el estudio de Gil-Lora, *et al.*, de 76,7% ^36^.

En las últimas décadas, el aumento de la deforestación, la urbanización, las migraciones e, incluso, de la pobreza, así como el acceso limitado a recursos sanitarios, han propiciado la aparición de enfermedades emergentes y reemergentes en el ser humano ^38^. Estos factores han contribuido a la expansión de los vectores y ha generado cambios en la epidemiologia de las enfermedades que trasmiten, como las rickettsiosis. Siempre se ha pensado que estas están asociadas a zonas rurales: no obstante, en el presente estudio, la frecuencia de IgG para rickettsias fue un poco mayor en personas procedentes de la zona urbana que de la zona rural (72,4 y 66,2%, respectivamente).

Estos datos difieren de otros estudios en departamentos colombianos como Antioquia, donde se reportó una mayor seroprevalencia en las zonas rurales, diferencia que probablemente se deba a que en dicho estudio la población estudiada pertenecía al Urabá antioqueño, región geográficamente distinta a la de Caldas ^36^. Estas variaciones en la frecuencia de anticuerpos pueden estar asociadas con las diferencias metodológicas de ambos estudios, ya que es posible que la frecuencia de anticuerpos contra el grupo de rickettsias de las fiebres manchadas sea mayor en zonas rurales, pues allí es más probable el contacto con garrapatas, cuya movilidad es restringida, en tanto que el contacto con pulgas puede ser igual en ambas zonas, ya que su presencia está asociada con las mascotas y los roedores ^39^^,^^40^.

En la población estudiada no se registraron diferencias significativas entre hombres y mujeres en la seroprevalencia, sin embargo, en la literatura especializada a nivel mundial se ha reportado una mayor prevalencia en los hombres debido a su mayor exposición recreativa y ocupacional ^37^^,^^41^^).^ En cuanto a la edad de presentación de la enfermedad, se ha reportado que la fiebre manchada de las Montañas Rocosas tiene mayor incidencia entre los 60 y los 69 años, y causa mayor mortalidad en niños menores de 10 años ^14^. En estudios en Antioquia se ha demostrado una seroprevalencia mayor frente a rickettsias de este grupo entre los 21 y los 40 años (40,38%), en tanto que, en los otros grupos etarios, estuvo entre el 28 y el 30 % ^42^. En el presente estudio, la menor seroprevalencia frente a este tipo de rickettsias se registró en el grupo de edad de 0 a 20 años (45,5 %), lo cual podría asociarse con su mayor interacción con animales, especialmente perros y gatos, portadores de diferentes tipos de ectoparásitos y, también, con su actividad laboral ^14^^,^^28^.

Algunos roedores como *Rattus rattus* y *R. norvegicus* se han considerado reservorios de *R. typhi*^28^; las zarigüeyas se consideran huéspedes y amplificadores de *R. rickettsii*^43^^,^^44^. Los bovinos, equinos y otros animales domésticos (perros o gatos) son sensibles a estos bacilos pero no son reservorios de rickettsias en áreas endémicas ^45^^,^^46^. En gatos y perros en Colombia, se ha logrado la identificación, no solo de los diferentes vectores (garrapata o pulga), sino también, de las rickettsias presentes en dichos vectores ^27^^-^^29^^,^^33^^,^^38^^,^^42^. En el presente estudio, el 66 % de los pacientes tenía mascota, especialmente perros; además, los pacientes refirieron tener contacto con bovinos (29%) o equinos (27%), en tanto que el 49,1% refirió la presencia de roedores en su domicilio o cerca de él. La frecuencia de anticuerpos contra rickettsias en estas personas fue alta, aunque el análisis de los datos no estableció una relación estadísticamente significativa de ninguna de estas variables con la presencia de rickettsiosis.

La inespecificidad de la sintomatología fue evidente en este estudio: los hallazgos sugestivos de la enfermedad, como fiebre, artromialgia, cefalea y erupción cutánea, se presentaron en los pacientes con seroconversión con una frecuencia similar a la reportada en la literatura ^7^^,^^36^. Los tres primeros síntomas también aparecen en otras enfermedades endémicas en nuestra región, como el dengue, el chikunguña, el Zika, la brucelosis o la leptospirosis, por lo que determinar la etiología exige que el médico tenga conocimiento de los principales signos y síntomas y la frecuencia de dichas enfermedades, así como que disponga de métodos de diagnóstico ^1^.

Debido a la inespecificidad de los síntomas, a la poca información epidemiológica que se tiene de las rickettsiosis y a la ausencia de métodos diagnósticos específicos, en la mayoría de los casos no se tiene en cuenta este grupo de enfermedades ^7^, lo que lleva a diagnósticos presuntivos asociados con enfermedades virales que no se tratan con los antibióticos útiles contra las primeras. La aparente ausencia de casos letales asociados con rickettsiosis en Caldas puede ser un indicio de la presencia de rickettsias del grupo de las fiebres manchadas menos patógenas que *R. rickettsii*^14^^,^^47^. Es necesario hacer pruebas con las muestras obtenidas y con otras rickettsias que se han descrito previamente en estudios en Colombia, tales como *R. parkeri, R. amblyommatis* y *Candidatus Rickettsia colombiensis*, entre otras ^38^^,^^48^^,^^49^, para establecer de manera indirecta la especie de *Rickettsia* asociada con las manifestaciones clínicas antes mencionadas.

Aunque la inmunofluorescencia indirecta es la prueba de referencia para el diagnóstico de rickettsiosis, también presenta serias limitaciones, entre ellas, la reacción cruzada con diferentes especies del género *Rickettsia*, cuya patogenia aún no se conoce en Colombia ^10^^,^^50^. Dado el tiempo que se requiere para establecer la seroconversión, ante la sospecha clínica de la rickettsiosis es necesario iniciar un tratamiento antibiótico empírico para evitar posibles complicaciones. Los resultados de la IFI pueden proporcionar al médico información que valide retrospectivamente la precisión de la sospecha diagnóstica clínica; además, dichas pruebas permiten conocer la epidemiología y el impacto en salud pública de las rickettsiosis en Caldas.

En conclusión, las rickettsiosis asociadas con los tres grupos estudiados no solo están presentes en el norte de Caldas, como lo han demostrado estudios anteriores, sino también en las otras zonas del departamento, lo cual responde a las características ecológicas de la región, su vocación agropecuaria y las características del hábitat de las personas.

## Archivos suplementarios

**Cuadro 1 suplementario.** Descripción de las variables clínicas, epidemiológicas y serológicas utilizadas en el cuestionario contestado por los pacientes atendidos en las instituciones prestadoras de servicios de salud del departamento de Caldas entre enero del 2016 y diciembre del 2019

Variable	Ítems evaluados
Variables sociodemográficas	Sexo Edad Procedencia Área Tiempo de residencia
Características de la casa	Bahareque Material Madera Otro, cuál
Presencia de mascotas	Perro Gato Otro, cuál
Contacto con animales, ratones y zarigüeyas	Presencia de ratas o ratones Presencia de zarigüeyas Contacto con bovinos Contacto con equinos Otro, cuál
Picaduras	Picaduras de pulga o Frecuencia Infestación con garrapatas o Frecuencia
Signos y síntomas	Fiebre Cefalea Erupción cutánea Artromialgia Hepatomegalia Diarrea Esplenomegalia Náuseas, vómitos Dolor abdominal Confusión Otros relevantes, cuál o cuáles
IgG contra *R. rickettsii, R. typhi* y *R. felis*	Primera muestra Positivo Negativo Título Segunda muestra o Positivo Negativo Título
